# Granulocyte-Colony Stimulating Factor Related Pathways Tested on an Endometrial *Ex-Vivo* Model

**DOI:** 10.1371/journal.pone.0102286

**Published:** 2014-10-02

**Authors:** Mona Rahmati, Marie Petitbarat, Sylvie Dubanchet, Armand Bensussan, Gerard Chaouat, Nathalie Ledee

**Affiliations:** 1 Equipe “Implantation et Dialogue Cytokinique Mère-Conceptus”, UMRS-976, INSERM, Université Paris Diderot, Hopital Saint Louis, Paris, France; 2 Service d′Assistance Medicale a la Procreation, Hopital Pierre Rouques - Les Bluets, Paris, France; Medical Faculty, Otto-von-Guericke University Magdeburg, Medical Faculty, Germany

## Abstract

**Introduction:**

Recombinant human Granulocyte-Colony Stimulating Factor (rhG-CSF) supplementation seems to be a promising innovative therapy in reproductive medicine, used in case of recurrent miscarriage, embryo implantation failure or thin endometrium, although its mechanisms of action remain unknown. Our aim was to identify possible endometrial pathways influenced by rhG-CSF.

**Materials and Methods:**

Hypothetical molecular interactions regulated by G-CSF were designed through a previous large scale endometrial microarray study. The variation of endometrial expression of selected target genes was confirmed in control and infertile patients. G-CSF supplementation influence on these targets was tested on an endometrial *ex-vivo* culture. Middle luteal phase endometrial biopsies were cultured on collagen sponge with or without rhG-CSF supplementation during 3 consecutive days. Variations of endometrial mRNA expression for the selected targets were studied by RT-PCR.

**Results:**

At the highest dose of rhG-CSF stimulation, the mRNA expression of these selected target genes was significantly increased if compared with their expression without addition of rhG-CSF. The selected targets were G-CSF Receptor (G-CSFR), Integrin alpha-V/beta-3 (ITGB3) implicated in cell migration and embryo implantation, Plasminogen Activator Urokinase Receptor (PLAUR) described as interacting with integrins and implicated in cell migration, Thymidine Phosphorylase (TYMP) implicated in local angiogenesis, CD40 and its ligand CD40L involved in cell proliferation control.

**Conclusion:**

RhG-CSF seems able to influence endometrial expressions crucial for implantation process involving endometrial vascular remodelling, local immune modulation and cellular adhesion pathways. These variations observed in an *ex-vivo* model should be tested *in-vivo*. The strict indications or counter indication of rhG-CSF supplementation in reproductive field are not yet established, while the safety of its administration in early pregnancy on early embryogenesis still needs to be demonstrated. Nevertheless, rhG-CSF appears as a promising therapy in some difficult and unsolved cases of reproductive failure. Indications of pre-conceptual rhG-CSF supplementation may derive from a diagnosed lack of endometrial expression of some target genes.

## Introduction

Recombinant Human Granulocyte-Colony Stimulating Factor (rhG-CSF) is used since late 1980's in haematological indications such as chemotherapy induced neutropenia [1], congenital agranulocytosis [2] or haematopoietic stem cell transplantation [3].

In reproductive medicine, rhG-CSF supplementation seems to be one of the most promising innovative therapies. Indeed, in distinct countries, rhG-CSF supplementation, either systemic (subcutaneous administration) or local (intra uterine infusion), is evaluated in the context of some unexplained recurrent miscarriages, repeated embryo implantation failures or thin unresponsive endometrium. Two randomised studies using rhG-CSF supplementation on IVF stimulation protocols, in case of repeated miscarriages, suggest a higher live birth rate and fewer cases of pregnancy loss [4] [5]. Moreover, rhG-CSF supplementation is tested in preliminary IVF protocols involving patients with a history of repeated embryo implantation failures (IF) [6]. There are also cases of pregnancy reported after intra-uterine infusion of rhG-CSF, in patients with IVF failure with endometrial trophic defect [7] [8] [9] [10].

However, the mechanisms of action by which rhG-CSF would positively influence the embryo implantation are largely unknown. The objective of the present study was to identify the possible relevant pathways influenced by a local administration of rhG-CSF, involving target genes pre-selected from a previous large scale microarray [11], using an ex-vivo model of endometrial microhistoculture [12]. In this model and considering the selected target genes, rhG-CSF seems able to influence some endometrial expressions crucial for the implantation process. These molecules whose expression seems to be under G-CSF influence are involved in the endometrial vascular remodelling, the local immune modulation and cellular adhesion systems.

## Materials and Methods

### Patients

81 women were enrolled for this study. All patients were involved in Assisted Reproductive Technology (ART) programs and were less than 38 years of age. There was no statistically significant difference in age and BMI between the different groups. All patients were non-smoker. They all provided a written informed consent and this investigation was approved by our Institutional Review Board, Agence de la Biomedecine, under the trial registration number 2013-A00072-43.

17 patients were fertile control women, involved in ART because of male infertility. All had delivered after either intra-uterine insemination or ICSI within the two first attempts. They were evaluated at least six months after their delivery and before a new attempt. The mean age in this group was 33 years (from 29 to 35 years old). The mean Body Mass Index (BMI) was 23.8 (from 21 to 27.9).

19 patients had repeated unexplained embryo implantation failure (IF) [13] defined as an absence of pregnancy despite the transfer of 10 or more good quality embryos (fresh or frozen-thawed) over several cycles. All embryos had a fragmentation rate of less than 20% and were at least at the 4-cell stage by day 2 after IVF. The inclusion criteria also included a normal uterine morphology (excluding fibroma, polyps, thin endometrium, synechia, uterine malformation) after ultrasonography, hysterosalpingography and hysteroscopy, a normal karyotype, a normal hormonal reserve (FSH<10 mIU/ml, antral follicle count >9 follicles) and normal responses to the hormonal stimulation (with more than 8 oocytes retrieved per cycle). Normal endometrial trophicity was defined after ultrasonography by an endometrial thickness over 7 mm and an endometrial volume over 2.5 cm^3^ during luteal phase. In this group, the mean age was 31.7 years (from 28 to 35) and the mean BMI was 22.6 (from 19 to 29).

15 patients had a history of unexplained recurrent miscarriages (RM) [14] defined by at least three subsequent pregnancy losses between 6 to 12 weeks of gestation, remaining unexplained after uterine morphology evaluation (ultrasonography, hysterosalpingography, hysteroscopy), standard auto-immune exploration (antiphospholipid antibodies as lupus anticoagulant and anticardiolipin antibodies), evaluation of the thrombophilic activity (protein S, protein C, factor V, factor VIII) and exploration of genetic markers (parental karyotype). The mean age in RM group was 31 years (from 25 to 36) and the mean BMI was 20.6 (from 18 to 25).

30 other patients participated to our study. They were involved in a pre IVF cycle endometrial biopsy program (pre IVF “endometrial scratching” protocol). These endometrial biopsies were used for ex-vivo microhistocultures.

### Endometrial biopsies

Endometrial biopsies were programmed during a monitored natural cycle, 7 to 9 days after the ovulation surge (LH surge), during the hypothetical implantation window. These biopsies were performed with a standard Cornier pipelle (CCD Laboratories, Paris, France), collecting material from superficial endometrial layers in order to minimise sampling variations.

One part of the endometrial sample was formalin-fixed and paraffin-embedded for later histological study. We used a standard haematoxylin and eosin staining protocol on 3 µm thick sections for routine histological evaluation. Moreover, a histological endometrial dating, to classify patients as “in phase” or “out phase”, was performed according to the Noyes criteria [15].

Another part was collected in RPMI 1640 Glutamax medium for endometrial ex-vivo culture.

A third and last part of the endometrial sample was transferred into an RNA stabilizer solution (RNA Later, Qiagen, Courtaboeuf, France) for further RNA extraction.

### Endometrial microhistoculture

The endometrial microhistoculture model used in this study has been previously described by our team [12]. In this ex-vivo model, the cellular functionality and differentiation are preserved for five days. As a brief reminder, endometrial samples (1 to 2 mm^3^) were taken from biopsies collected in RPMI 1640 Glutamax medium, during the 2 or 3 first hours after collection. We put these endometrial blocks on collagen sponge gels (GelfoamH, Pharmacia Upjohn, Kalamazoo, MI, USA) which are placed into supplemented RPMI 1640 Glutamax culture medium. The medium supplementation was as follows: 15% heat-inactivated foetal calf serum, 1% penicillin and 1% streptomycin, non-essential amino acids, 1% sodium pyruvate (all from Gibco BRL, Life Technologies, France), 50 nmol/l of estradiol and progesterone (Sigma, St Quentin-Fallavier, France). Estradiol and progesterone supplementations were repeated daily. The cultures were maintained at 37°C in a 5% CO2 humid atmosphere.

For rhG-CSF stimulation, the five culture conditions were: rhG-CSF at either 20, 100 or 200 ng/ml, or G-CSF blocking antibody (anti G-CSF) at 3 µg/ml (all recombinant proteins and antibodies from R&D Systems, Lille, France). Recombinant protein or antibody was added to the culture medium every day during three consecutive days. After a three days incubation, the endometrial samples were collected in a RNA stabilizer solution (RNA Later, QIAGEN, Courtaboueuf, France) for RNA extraction and RT-qPCR, as described in the corresponding paragraph.

To specifically target the rhG-CSF endometrial action, the same experiments were performed with Granulocyte-Macrophage-CSF (rhGM-CSF) at either 20, 100 or 200 ng/ml, or GM-CSF blocking antibody (anti GM-CSF) at 3 µg/ml (recombinant proteins and antibodies from R&D Systems, Lille, France).

The biopsies from 30 patients, involved in a pre IVF cycle endometrial biopsy program, were used for these ex-vivo microhistocultures. Biopsies from 17 patients provided 57 endometrial samples (1 to 2 mm^3^) for rhG-CSF microhistocultures. Biopsies from 13 patients provided 39 samples (1 to 2 mm^3^) for control rhGM-CSF microhistocultures.

Every patient was her own control, e.g. for each stimulation condition, an endometrial sample from the same patient was concomitantly placed in identical culture conditions, without the corresponding recombinant protein or blocking antibody. For these control cultures, only estradiol and progesterone were daily added (EP condition), as described, during the three days incubation period. Therefore, for the same endometrium, we could compare the target gene expressions under specific stimulation and EP condition.

### Endometrial RNA extraction, quality control and cDNA synthesis

The total endometrial sample was homogenized with an Ultra Turrax T15 (IKA-WERKE) and the homogenate was then purified on Qiashredder columns (Qiagen, Coutaboeuf, France). The total RNA was isolated with the Qiagen RNeasy mini kit (Qiagen, Courtabeuf, France) including the RNase-free DNAse set. Recombinant RNase inhibitor (10 units/µl of extracted RNA) was added to prevent RNA degradation. RNA quantity and quality were confirmed by the analysis with an Experion system and RNA Std Sens analysis kit (Bio-rad, Marnes la Coquette, France). The RNA was then stored at −80°C. The RNA (1 µg) was first reverse-transcribed into cDNA using random primers and SuperScript III reverse transcriptase (Invitrogen, Carlsbad, CA, USA) following the manufacturer's instructions.

### Target genes selection and real time quantitative PCR (RT-qPCR)

Hypothetical pathways and molecular interactions putatively regulated by G-CSF were selected on the basis of gene expression deregulations according to the indications stemming from a previous large scale endometrial microarray study [11]. Briefly, this microarray analysis suggested pre conceptual extensive endometrial deregulations in patients with IF or RM. When analysing those intricate pathways involving G-CSF, the expression of some gene seemed strikingly specifically deregulated. Selecting among these highly deregulated genes, those hypothetically depending on G-CSF action, we picked up those molecules involved in immune regulation, coagulation system or integrins. We therefore selected the following genes, suggested as G-CSF targets in the endometrium: G-CSF Receptor (G-CSFR), Integrin alpha-V/beta-3 (ITGB3) known to be implicated in cell migration and embryo implantation, Plasminogen Activator Urokinase Receptor (PLAUR) described as interacting with integrins and implicated in cell migration, Thymidine Phosphorylase (TYMP) implicated in local angiogenesis, CD40 and CD40 Ligand (CD40L) involved in cell proliferation control.

Specific primers for these targets genes and Ribosomal Protein L13A (RPL13A), as the reference gene, were constructed using the Universal Probe Library Assay Design Center (\.rocheapplied-science.com) and their sequences were searched against Gen-Bank sequences with the BLAST program to ensure the specificity of primers. Real-time PCR was carried out using a LightCycler 480 apparatus (Roche, Meylan, France). Reactions were set up using the following final concentrations: 0.5 mM of sense and antisens primers, 1X 480 SYBER Green master mix and 4 ml of 1/20 diluted cDNA. Cycling conditions were as follows: denaturation (95°C for 5 min), amplification and quantitation (95°C for 10 s, 60°C for 10 s and 72°C for 15 s) repeated 40 times, a melting curve program (65–95°C with a ramp rate of 2.2°C/s) and a cooling step to 4°C. In addition to the no-reverse transcription and no-template controls, an independently inter run calibrator (IRC) was included in each RT-PCR assay. This IRC was obtained from blast-cells. In each assay, an aliquot of the IRC cDNA was 20 times diluted and was submitted to the qPCR protocol as the unknown samples. PCR efficiencies for each quantified target and reference were calculated using known serial dilutions of each specific cDNA. Data were analysed using the LightCyclerH480 Software release 1.5.0. Each specific target transcription level was normalized to the geometric mean of the transcription levels of the reference gene, using the Advanced Relative Quantification of the LightCyclerH480 Software. Efficiency was controlled for each specific gene amplification.

For each sample, the results are expressed in concentration ratio (target gene mRNA level/reference gene mRNA level). For microhistoculture samples, these ratios are compared between the stimulated culture condition and the EP basal condition, as described in the dedicated paragraph, each patient being her own control.

### Statistics

The Wilcoxon test was used to compare target genes mRNA expressions in each patient group and in each culture condition. The Spearman test was used to search for a correlation between different gene expressions. The statistical assessments were performed using the Stat View software (Abacus Concepts, CA, USA). The significance level was set up at p<0. 05.

## Results, Discussion, and Conclusions

### Results and discussion

**Figure 1:**
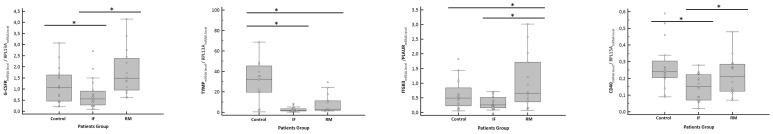
Endometrial mRNA expressions variation in control, IF and RM patients. Figure 1a: G-CSFR mRNA expression variation in control, IF and RM patients. Figure 1b: TYMP mRNA expression variation in control, IF and RM patients. Figure 1c: ITGB3 and PLAUR mRNA expressions variation in control, IF and RM patients. Figure 1d: CD40 mRNA expression variation in control, IF and RM patients. IF: Implantation Failures. RM: Repeated Miscarriages. Results expressed in concentration ratio (Arbitrary Units) between target gene mRNA level and reference gene mRNA level. (*) Statistically Significant Difference, p<0.05.

**Figure 2:**
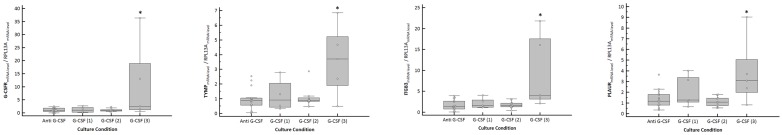
Endometrial mRNA expressions variation after rhG-CSF stimulation. Figure 2a: G-CSFR mRNA expression variation after rhG-CSF stimulation. Figure 2b: TYMP mRNA expression variation after G-CSF stimulation. Figure 2c: ITGB3 mRNA expression variation after G-CSF stimulation. Figure 2d: PLAUR mRNA expression variation after G-CSF stimulation. Anti G-CSF: 3 days culture with G-CSF blocking antibody daily supplementation at 3 µg/ml. G-CSF (1): 3 days culture with rhG-CSF daily supplementation at 20 ng/ml. G-CSF (2): 3 days culture with rhG-CSF daily supplementation at 100 ng/ml. G-CSF (3): 3 days culture with rhG-CSF daily supplementation at 200 ng/ml. Results expressed in concentration ratio (Arbitrary Units) between target gene mRNA level after culture with and without specific stimulation, each patient being her own control. (*) Statistically Significant Difference, p<0.05.

Both the protein production and mRNA expression of **G-CSF receptor (G-CSFR)** have been shown to be localised at the maternal foetal interface since the late 1980's [16], with cyclic and gestational regulations [17] [18] and a trophoblast growth promoting role [19]. When considering the G-CSFR mRNA expression in control vs. infertile patients, we did not notice any significant global difference. But when considering the IF subgroup within the infertile patients, the G-CSFR mRNA expression was significantly lower (p = 0.01), as shown in figure 1a. In the ex vivo model, we observed a significantly higher mRNA expression at the highest dose of rhG-CSF stimulation (p = 0.01). We did not find any significant mRNA variation with the adjunction of anti G-CSF (figure 2a).

**Thymidine Phosphorylase (TYMP),** also known as the platelet-derived endothelial cell growth factor (PD-ECGF) or gliostatin, is a key angiogenesis promoting enzyme [20] as well as a cell migration promoter, especially by modulating integrin expression [21]. Its presence is also described in the endometrium [22]. In our infertile group, the TYMP endometrial mRNA expression was significantly lower (p = 0.0019), in those patients with either IF or RM (figure 1b). When the cultures were stimulated with the highest dose of rhG-CSF ex vivo, the TYMP endometrial mRNA expression appeared significantly higher (p = 0.04), as illustrated in figure 2b. G-CSF blocking antibody had no significant action on TYMP expression.

**Integrin alpha-V/beta-3 (ITGB3),** which is present in the endometrium [23], is described as being highly implicated in the cell migration and embryo implantation process [24]. It is a major endometrial adhesion molecule described as such not only in Human, but also in various animal models such as bovine [25] [26], porcine [27] [28] and ovine [29]. An ITGB3's down regulation has also been incriminated in pathological situations involving an impaired endometrial receptivity [30]. As an expected G-CSF endometrial target, ITGB3 mRNA expression was significantly enhanced (p = 0.001) by application, of the highest dose of rhG-CSF ex vivo (figure 2c), and without any change despite anti G-CSF adjunction. However, in this study, we could not obtain a significant difference in ITGB3 endometrial mRNA expression between control and infertile patients, either with IF or RM. We thus tried to consider the possible interaction of ITGB3 with PLAUR.

**Plasminogen Activator Urokinase Receptor (PLAUR),** or uPAR (Urokinase Plasminogen Activator Receptor), is an essential actor in tissue remodelling through cell migration, proliferation and survival [31]. PLAUR is localised in the endometrium, its expression varying during the menstrual cycle [32]. It is described in early placental bed pregnancies and especially in those immune endometrial cells called uterine Natural Killers [33]. PLAUR is often implicated in impaired trophoblastic invasion, leading to pathological pregnancies with intra uterine growth retardation or pre eclampsia [34] [35] [36] [37]. Moreover, the PLAUR signalling pathway is described as requiring integrins, including ITGB3 [31]. Therefore, since we could not find any significant difference in endometrial PLAUR mRNA expression between control and infertile patients, we studied endometrial PLAUR and ITGB3 interaction by considering the following ratio: ITGB3 mRNA level/PLAUR mRNA level. When considering the ITGB3/PLAUR mRNA expressions (figure 1c), we observed a significant endometrial disequilibrium in these infertile patients with a history of RM (p = 0.02). When stimulated with the highest dose of rhG-CSF, PLAUR endometrial mRNA expression was significantly enhanced (p = 0.04), as illustrated in figure 2d. Again, these variations were not observed when adding anti G-CSF.

**CD40 and its ligand CD40L** are membrane molecules involved in the control of cellular proliferation via regulation of apoptotic mechanisms [38]. They are localised on immune cells, haematopoietic progenitors, epithelial cells and carcinomas [39]. When considering our previous microarray study, these genes seemed highly deregulated in the endometrium of infertile patients, and hypothetically interacting with G-CSF. In this study, CD40 endometrial mRNA expression did not significantly vary between control and infertile patients, when taken globally. But when considering the IF subgroup within the infertile patients, the CD40 mRNA expression was significantly lower compared to control group (p = 0.04), as shown in figure 1d. In the ex-vivo model, at the highest dose of rhG-CSF stimulation, CD40 mRNA expression only tend to be higher, without reaching significance (data not shown). CD40L mRNA expression level was very low in either control or infertile patients and did not vary in the ex-vivo culture with rhG-CSF supplementation. For both genes, we did not observe any significant mRNA variation with anti G-CSF adjunction.

The mRNA expression variations for G-CSFR, TYMP, ITGB3 and PLAUR, observed under rhG-CSF stimulation, were found to be correlated, as shown in table 1, suggesting that the described pathways may be intricated.

**Table 1:** Correlation between target genes mRNA expression under G-CSF stimulation.

*Correlation between Target Genes Expression*	*Rho*	*p*
**ITGB3 & TYMP**	31%	0,05
**PLAUR & ITGB3**	47%	0,003
**PLAUR & TYMP**	45%	0,005
**GCSFR & ITGB3**	67%	<0,0001
**GCSFR & TYMP**	46%	0,006
**GCSFR & PLAUR**	37%	0,02

To specifically study the action of endometrial rhG-CSF on its selected target genes, we used the same ex-vivo experiments with rhGM-CSF supplementation during 3 consecutive days. This control supplementation did not induce any variation on mRNA expressions of G-CSFR, TYMP, ITGB3 or PLAUR, as observed with rhG-CSF adjunction.

In all cases, adjunction of either anti G-CSF blocking antibody or anti GM-CSF blocking antibody did not show any variation in the target genes expression with the present ex vivo model. This may be due to existing redundant pathways or linked to the type of selected blocking antibody.

### Conclusions

After showing the difference of expression of some hypothetical endometrial targets in infertile patients, and after observing the variations of expression of these target genes under specific rhG-CSF stimulation ex vivo, this study illustrates the putative key role of G-CSF during the embryo implantation process. This cytokine seems able to modulate fundamental genes intervening in the local embryo adhesion, cell migration, tissue remodelling and angiogenesis, unavoidable for a successful implantation and further placentation.

RhG-CSF appears actually as a promising innovative therapy in some difficult and unsolved cases of reproductive failure. However, strict indications of this supplementation in reproductive field are not established yet. Such expected endometrial actions may be tested after in vivo rhG-CSF supplementation and safety of such treatment still needs to be demonstrated on early stages of embryogenesis. Specific indications of pre conceptual rhG-CSF supplementation may also derive from diagnosed lack of endometrial expression of some of its target genes.
